# Extra-Limites

**Published:** 1832-10-01

**Authors:** 


### EXTRA-LIMITES.

### LETTER

FROM

### DR. HACKET TO DR. JOHNSON,

RESPECTING

### DR. STEVENS' PUBLICATION.

Sir,

I shall once more trouble you, in consequence of Dr. Stevens' reply to my statement, published by you; and ere I proceed to that subject, I must first express my surprise at the even *more than* insinuation he expresses of a combination entered into between yourself, some others, and me, against him—but there is a hardihood of assertion, a real "Corinthian" effrontery about this gentleman, rarely to be met with in the common walks of life; but which, when found amongst those who, by profession or situation, rank as gentlemen, calls forth, according to circumstances, either surprise or indignation, or possibly both.

It is not necessary for me to declare to you, but it becomes necessary from so foul an insinuation, to say, that you are totally and entirely unknown to me, except in your public professional character; and that no communication directly or indirectly ever passed between us until I addressed to you the paper for publication which you were good enough to give to the public. The natural disgust arising in a candid and ingenuous mind from a plain statement of facts upsetting the tenets of a most vain and presumptuous man has, it seems, been sufficient to call forth his ire and wrath against you—this you will know how to appreciate, recollecting that the abuse of some people is perhaps the greatest praise that any individual can receive. I am not personally acquainted with Dr. Stevens, nor is it *now* possible I ever shall be; nevertheless he has depicted himself in such glowing colours that I now know him perfectly well, and know too how to estimate his full value,—his candor and fairness are equally appreciated, since my statement, by the generality of practitioners throughout the West Indies, as by myself.

In looking over the reply offered to the public, I feel, I confess, a mingled sensation of pity, derision, nay, perhaps I might add, contempt for the exhibition of the unbridled arrogance and presumption displayed therein, and the want of taste and gentlemanly feeling depicted throughout the whole, from first to last; but checking all such sensations, and coming to particulars, the following seems the claim set up by Dr. Stevens on his own behalf, divested of all that embellishment or special pleading, so distinguishable throughout, and the consequent accusation contained therein against us.

First:—He insists that we, the medical officers of the garrison of Trinidad, are solely indebted to him for the success we obtained in the treatment of fever there, because we had a great mortality previous to his visit in the middle or latter end of August, 1828; which mortality, he *insists*, ceased from "the moment" of his arrival: having it seems pointed out to Mr. Greatrex the errors of the previous practice followed, recommending in lieu thereof a combination of muriate of soda and nitrate of potash to replenish the colouring and saline properties of the blood; to the former of which, in common with "the waters of the deep" it owes its preservation, but which becomes either exhausted in the course of fever, thereby causing its dissolution, and consequent death of the individual; or is, in point of fact, the only and real cause of disease or death; and which recommendation, from our own shewing, was not only complied with, but generally adopted.

Secondly:—That, even from our own shewing, we have charged him wrongfully with misrepresentation and want of candor; for, in point of fact, there is no difference between the anatomical description given by us of those who had died of tropical fever, and that published by him in his "Observations on the Blood;" and so far from exhibiting any want of candor, that Mr. Greatrex's letter fully bears the learned gentleman out in the pretension set forth by him, that from adopting "his system" success immediately followed; for there was no other that would have caused the great diminished mortality from fever in the Royal Regiment; and that, if we were possessed of any other curative process we were criminal in not having employed it previous to his visit.

Thirdly:—We are accused of not only misunderstanding his views altogether in "talking of soda or alkalies" in the treatment of fever, which so far from recommending, he disapproves of being administered at all: considering all such the reverse of beneficial in fever; that we also prescribed supertartrate of potash, which he most highly disapproves of, considering its excess of acid as acting most injuriously on the stomach or system generally in fever—that we used the hot-bath at the times or stages of fever when we should not—in fine, that we mixed up his remedy without understanding his views or hypothesis, with every thing we should not, and even with most of those remedies which he considers injurious and destructive, and which he asserts should not have been given (oh! wonder-working remedy to cure after all) to the patients, but which, notwithstanding we did wilfully and maliciously, it would appear, administer to them, mark, reader, *for the sole purpose of depriving* him of the eclat so justly his due for the cure of fever at Trinidad!

At first glance, these may appear to some grave and heavy charges; they are made with a facility and self-complacency which leaves us at a loss whether to admire most the hardihood of assertion, or the arrogance of the individual who could have made them. Now as regards the first pretension quoted of Dr. Stevens, and his demand to what other source we owed success, but the system he recommended, and to the assertion that "the mortality of the garrison ceased from the moment of his arrival at Trinidad," I must now say, that *both his pretension and assertion are equally unfounded;* and in reference to the conversation, he states, that he held with Mr. Greatrex, at St. James's Hospital, I must be permitted to doubt the accuracy of the Doctor's report of it in the following quotation—"Dr. Hacket states, when I visited the hospital at Trinidad, I admitted the similarity of their practice to my own. Now this is a mistake, for I knew from the rate of the previous mortality that there must have been some radical error in their practice; I saw clearly what this was and pointed it out to Mr. Greatrex." And again,—"from the beginning of that year, and up to the middle of August, it is a fact that the Royals alone lost in that hospital upwards of forty men from fever; it is now generally, I believe, admitted, that the blood is a living fluid, and if so it must then, like every thing else that possesses life, be subject to disease. It is also true that the mortality ceased only from the moment that I had not only directed their attention particularly to the circumstance, that in the fever which they had to contend with, the diseased state of the blood was the chief cause of death, but pointed out the means by which they might greatly lessen the mortality by preventing those fatal changes in the vital current, which are in reality, in these fevers, the great cause of death. It is also true that, from that period they scarcely lost even a case of fever, except the three men already mentioned, which men had been beyond all redemption before they were admitted, and the other two men who died on the very day they were brought in. But I know well what their practice was both before and after the middle of August, 1828, and I know the result. I know also what these unnecessary measures were which I had not recommended, and which they unnecessarily combined with the saline treatment, apparently for the purpose of taking to themselves as much of the credit as they possibly could. They knew however of all these other measures to which they allude, previous to my visit, yet they allowed the mortality to continue up to that very period; but I shall afterwards prove, that some of these measures were too insignificant to have had any effect on the result while others, but particularly the hot-bath, were not merely inert, but actually improper, but above all when employed at the period at which they used it."

As to the first quotation, viz. that there must have been some radical error in the practice, and that he pointed it out to Mr. Greatrex—this appears to me exactly on a par with the general tone and hardy assertion, that Dr. Stevens never hesitates to indulge in; yet all which I am compelled to say, in point of fact, is *utterly unfounded*,—it is *utterly*

*impossible that any such* observations as he states could have been made by him to Mr. Greatrex—because, in the first place, had *such* been *actually* made it would undoubtedly have been stated to me the following day, when Mr. Greatrex informed me of the learned gentleman's hypothesis of an acid in the blood! He assuredly then would have informed me of those errors in our practice pointed so "clearly out" to him, instead of telling me that Dr. Stevens approved, (notwithstanding all he now asserts to the contrary) highly of our practice, and was struck with the similarity of it to his own, and which, by the bye, en passant, I would now have the learned Doctor to know, I consider no compliment; at all events, as regards the conversation, I can only say for myself, these errors of practice, if they be errors of practice, (for the ipse dixit of Dr. Stevens *now* can have but little weight with practical men) were never stated to me:—again, I say, it was *utterly* impossible for him to have pointed out to Mr. Greatrex the errors of that practice which produced the previous mortality; for *there was no record of it shewn to him;* this did not arise from design but from mere accident, for it so happened, that the registers of our practice shewn to him only embraced the period of Mr. Greatrex's being in charge; he having joined the garrison but a few weeks before. And it will be seen by the following extract from General Orders, that Mr. Greatrex had not been previously at Trinidad, and consequently had nothing whatever to do with the previous practice, the errors of which, though "so clearly seen," it seems by the intuitive talent and vivid imagination of Dr. Stevens, for he had no other data *whatever* to go on, could not therefore, I say, be pointed out to Mr. Greatrex, either as regarding the past or as immediately affecting himself; and further, although the learned gentleman, with his accustomed hardihood, asserts otherwise, yet I solemnly declare, with the exception of using his nostrum, and which I now regret I ever sanctioned being used, for *how* highly held in estimation will hereafter be stated, there was no deviation whatever in our practice, though "its errors had been so clearly pointed out" since first fixed and permanently established about the middle of July, 1828, to the remotest period of our continuance at Trinidad, notwithstanding all Dr. Stevens asserts to the contrary, though absent himself some thousands of miles from its application.

*Head Quarters, Barbadoes,* 17*th June,* 1828.

"No. 7, Assistant-Surgeon Greatrex, of the Royal Regiment, will proceed from Barbadoes by a similar conveyance to Trinidad."

Mr. Greatrex, in compliance with the above order, joined about the 24th of June; it will therefore be seen, he could not have been at Trinidad but a very short period previous to Dr. Stevens' visit there, in the middle or latter end of August of the same year.

Having now, I trust, fully and clearly shewn the fallacy, to give it the mildest term, of Dr. Stevens's statement, as to the "errors of practice, and his pointing them out," I have next to remark on the assertion he makes, that the mortality of the Royal Regiment ceased "from the moment of his arrival at Trinidad:"—this is a vain and empty boast, and exactly in keeping with the general highly coloured portrait Dr. Stevens so constantly indulges in of himself. It appears from the returns of the Royal Regiment, recently taken from the hospital books, and examined by me at St. Lucia, that the strength of the corps, rank and file, was about 350; that the admissions into hospital, (from December, 1827, the time of their arrival in Trinidad, to December, 1828) of remittent fever cases alone, amounted to 942; the deaths from the same 43: from this it will appear, that every man in the regiment, within a year, averaged nearly three attacks of malignant fever; consequently those of the most weakly and broken down constitutions, from previous excess, died, whilst the surviving got every day more seasoned or acclimâted to the island, as usually happens to all corps; and therefore it is that the subsequent attacks of fever come down every day more to the standard of the milder form to be met with amongst the troops themselves, and very different from the intense and malignant form to be met with in first comers, or Europeans:—but as to the details of the events of the memorable 1828; the admissions into hospital from the 20th of June to the 20th of September, the finale of the Quarter, and which was one of the most rife in sickness we had experienced, amounted to 258 cases of remittent fever; the mortality amongst them was very great, amounting to twelve, exceeding by one half the death-roll of the preceding quarter; this mortality ceased on the 20th of July. From this period to the middle of August we lost *but one case* of this fever: however, in the succeeding quarter, that is from September to December, we lost five cases, the febrile admissions for the same period amounting to 224; though, let it be borne in mind, *the quoted letter* states we had not lost *one man within this said period*—yet five we lost, though Dr. Stevens, it must not be forgotten, towards the end of the *preceding* quarter, had kindly shed, by his own account, the *unassuming* mantle of his mighty wisdom over us, and "lightened our darkness" as to our future treatment; claiming, therefore, the merit of a blank death-roll for September, *following* his visit immediately; and would, I dare say, claim the same for the month of May *preceding*, in which we lost no fever case, had the light of his countenance happily shone on us or the garrison at that time. From the foregoing it will be seen, that the mortality in the Royal Regiment ceased a *month previous* to Dr. Stevens' visit to Trinidad, and not from *the moment* of his coming there, though with his usual *diffidence* he asserts the reverse; and this success commencing a month before, continued almost uninterruptedly for a month after his departure; though the succeeding quarter, as already stated, proved, it will be seen, a fatal one. Thus is shewn, that for a certain period previous to the learned gentleman's visit, and let it be borne in mind too, in the very heart of the most unhealthy period of the year, we had not lost a man from remittent fever; and after his visit it so happened, that some of those who died, were those in which his combined soda plan was tried. I do not mean to insinuate, they died from the use of this remedy, for though prescribed as an addenda, *our own established* "system" was not omitted; for, in truth, from first to last, this compound was never considered more than a mere secondary remedy, by either of us,—it was so, at least, considered by me; and if Mr. Greatrex, (who I am sure did not) entertained other sentiments, I was not aware of them: indeed, to shew the perfect indifference, or rather, let me say, the disrespect, I myself entertained for the learned gentleman's remedy or doctrines, whether it was an acid in the blood, which I firmly believe, at first, made a part or parcel of his hypothesis, when he mentioned it to Mr. Greatrex, and which, that gentleman's letter bears evidence to—or the renovation of its saline impregnation, which he now adopts; be it however which it may, at best it is of little consequence. I perfectly remember jeering Mr. Greatrex on the infallible "sodaic mixture," reminding him, the first deaths we had had for some time, was when we deviated at all from the system of practice we had established, by the introduction of any auxiliary; and though Dr. Stevens indulges in his usual hyperbole, for which, indeed, he seems to have "the bump," about some of these men having been conveyed to the hospital "under a burning sun at mid-day," yet forgets to mention, their barracks were not above an hundred yards, or thereabouts, from the hospital, and consequently, the exposure, if they really were so exposed, which I much doubt, as their comrades are always kind and attentive to the sick, must have been very trifling, and although so pathetically portrayed, yet I imagine, even if it were as described, it would not be admitted by any tropical practitioner, who was really a practical, and not merely a hypothetical man, as a just and proper reason for not removing a sick man in fever, when the necessary promptness and activity too, in his treatment, is considered, and when the hospital and barracks of St. James's and the Artillery barracks approximated so immediately to each other. And here let me observe, whilst Dr. Stevens can find an easy apology for those dying to whom this remedy was administered, viz. "three being beyond redemption, and two dying the day of their admission into hospital." Is it not strange, too, when he talks so arrogantly of the "previous mortality" in the Royal Regiment, that it never struck him, a similar apology, did we deem any requisite, might also be adduced for that said mortality, different in no respect, in a great number of instances, from those five cases he mentions? Had he less self-sufficiency, might not this also elucidate for him, ere he gave way to vain boasting, the "fact that the Royals alone in that hospital lost upwards of forty men from fever," many of whom were beyond redemption, nay, even died on the very day of their admission; and yet, after all, be it known, even that mortality amounts to little more, at a rough calculation, than four per cent of the admissions, which I have already stated to be 942. By the bye, I cannot omit noticing here, another instance of the hardihood of assertion, though generally founded on some paltry quibble, that Dr. Stevens indulges in, when he declares, "not one of these men ever had a particle of soda administered to them;" when he ought to have known, particularly when he "saw so clearly our errors," or from Mr. Greatrex's letter, or from what was related by me, that either the carbonate or subcarbonate of soda, in solution with toast-water, was the common beverage, *ad libitum*, for every fever patient in hospital.

To revert again to the vaunted death-list of the Royal Regiment having been so great *before*, and so small *after* the Doctor's visit; and as great stress is laid by him on this point, as conclusive evidence in his favor, the answer is plain: the corps had but the last of the preceding year, landed in a sickly island from Barbadoes, and had to undergo what is emphatically though vulgarly called "a seasoning," sufficient, it will be admitted by those who know the tropics, to account for a great mortality; but, alas! for the credit of the profession generally to relate, another cause I fear existed : willingly, *most willingly*, would I cast this in oblivion, but (I cannot help saying it) the almost unprecedented assurance of Dr. Stevens—unprecedented, at least, among gentlemen, and foreign to the feelings or conduct of all those civil or military I have been in the habit of associating with—and above all, the wish to guard the public by a fair and explicit statement, from his representation, however repugnant to my personal feelings, and I confess it is painfully so, now force the detail from me. The Assistant Surgeon of the Royal Regiment, who accompanied it to Trinidad, and was in charge of the corps there, had long given me great dissatisfaction, to say the least of it, by the slovenly manner the sick were generally attended to, as well, indeed, as by the way the whole hospital duties were performed,—drunkenness, almost habitual, was the cause; this I long suspected, and as soon as a fair opportunity of prosecuting it to conviction occurred, charges were preferred by me for that offence, and consequent neglect of the sick—he was tried for the same, and dismissed by his Majesty from the Service, with comments on the degree of his offence: his immediate successor, sent to take charge of the regiment, most unfortunate both for the garrison and myself, proved even worse, and more habituated to drunkenness than the former,—most unfortunate, I say, for the well being of the regiment, in having such a character in charge of it, and in giving me a disagreeable degree of notoriety throughout the whole command, being obliged, in justice to the sick and the service, to place him also in arrest, and prefer similar charges as in the former case; previous, however, to the assembling of the Court Martial which was ordered, he died from intoxication. It is well known to all conversant in West India fever, *minutes*, comparatively speaking, are *hours*, as regards the application of our remedies. Notwithstanding I gave all the attention to the hospital my other avocations permitted, yet from such characters as the foregoing, it is evident the sick could not have had all that ready and watchful attention so peremptorily required in a tropical climate, rendering themselves incapable either of prompt attention, or the use of their faculties, from frequent intoxication; and, most strange it is to say, yet any one conversant with military affairs will know the fact, how long such a state may exist in those who have charge of an hospital, without a single individual connected with it, or even the sick inmates themselves exposing the fact; nay, they would rather, on the contrary, conceal it from those who might and would take cognizance of it. Are not these sufficient reasons, let me ask, to account, in some, at least, if not in a great measure, connected with all I have previously stated, for the mortality in the Royal Regiment, without adverting at all, as a contrast, to the wonder-working effects of Dr. Stevens' "sodaic mixture" afterwards? Let it be also borne in mind, *our successful system* was established previous to the so much boasted "*veni, vidi, vici*," visit of Dr. Stevens to Trinidad.

When I contemplate the foregoing, I acknowledge there does seem to me something *exceedingly* ridiculous, in being necessitated thus to come to actual detail, and be obliged to particularize every minute circumstance, to repel the unfounded pretensions of such a practitioner, who doubtless might have come, as he states, in consequence of hearing, that fever then prevailed at Trinidad; though this ostensible cause, the gentleman himself, or those who accompanied him, did not then give out; and if such had really been the motive of his excursion, one would have thought he would have *made a point* of seeing me, the then principal medical officer, and not be content with a mere visit to the hospital. But divesting his statement of what I cannot help deeming *flim flam*, the fact was, a Danish brig of war, as they are in the habit of doing, came on a cruise to the Gulf of Paria; Dr. Stevens availed himself, as it was then said, of the opportunity of visiting the island, both for recreation and pleasure, and accompanied her. I hate any thing like illiberality, and love my profession too well to attempt to shut the door of any military hospital, under my control, against any practitioner who might desire to visit it; on the contrary, I have *most gladly* always received those who were pleased to come to our hospitals, shewing and explaining every thing connected therewith; but, I confess Dr. Stevens's conduct has a tendency, at the present moment, to engender doubt and suspicion in my mind towards all such visitors in future.

Ere I take leave of this part of my subject altogether, I have to state that although, in conformity with Mr. Tegart's recommendation, croton oil *was always* more or less *used* in the hospital, yet I confess I was blinded by prejudice, and little encouraged to its more extended use by the professional men I spoke with on the subject. So strong was this prejudice, that, instead of applying the remedy in the fair and regular progress of fever, which I should have done, it was, I now deplore stating, never, perhaps, administered but as "an awful remedy," in fact a dernier resort, "when hope had fled," and when fatal symptoms had already exhibited themselves—*then*, and *not till then*, was this most invaluable oil prescribed. The mortality, however, that took place in the early part of July, 1828, and its extraordinary effects in one or two particular cases around us, leading to a more early exhibition than we had hitherto been wont of this most powerful febrifuge, the effects produced, without any exaggeration, were really astonishing—so much so, as to daily embolden us more and more in the exhibition of it, either in large and repeated, or more frequent small doses, and in enemata also; so that, from sheer practical experience of its effects, greatly heightened, as we afterwards discovered, when in combination either directly or alternating with large doses of quinine, we imagined this remedy approached nearer "a sovereign one" in tropical fever than any other, be it what it may, that ever has as yet been adminisrered in any disease. I say not this at random; I state it, I repeat, from sheer practical experience of the really wonder-working effects produced by it in the most malignant form of yellow fever—effects were frequently noticed both by the serjeant and orderlies, and indeed the servants generally of the hospital, so remarkable and striking were they. Corroborative of what we really attributed our success to, independent of those letters I forwarded to you from many of the practitioners at Trinidad, and as proof of what we considered our chief stay in tropical fever, the following extract of a letter of mine to Sir James M'Grigor, relative to the state of the garrison at Trinidad, will, I trust, be deemed a sufficient refutation to the statements of Dr. Stevens, that to disguise the advantages we obtained from his recommendation, and to deprive him of the eclât his just due, we mixed up his remedy with "useless," "inert," (croton oil and quinine useless and inert!) or even prejudicial ones, to secure to ourselves a merit we did not deserve!! I forbear any comment on the spirit that would engender *such* an idea, and envy not the moral constitution of that mind which could cherish, much less give utterance to, such thoughts;—and, in reference to the letter alluded to, let it be borne in mind, when I wrote that letter I had not the slightest expectation of any claim being about to be set up by Dr. Stevens, or indeed any other person, and it will still serve to prove *how* little, in the curative plan of treatment, his saline medicine was thought of, by its being perfectly unnoticed by me, when, too, I could have no possible motive for concealment—at least none of the exceedingly base and unworthy motives attributed to me so unreservedly by the *elegant* and learned author of the saline doctrines. Indeed, I would appeal with confidence to all my confrères who know me, or with whom I have ever done duty, if they ever noted in me the *slightest* inclination to withhold the meed of praise from any individual, civil or military, be he who he might, from whom I derived a practical hint;—yet a person, totally unknown to me, and to whom I am totally unknown, hesitates not to impugn my motives, and publicly charges me with the basest and vilest intentions. But, to return from this digression, the following is the extract alluded to in the forgoing, dated Trinidad, August 10th, 1829.

"I avail myself of this opportunity to tell you we have had a severe invasion of tropical fever at St. James's, commencing about the 30th of July, of which, too, the shipping in the harbour partake; since when our admissions have been numerous, and some of them of the most intense character I have ever witnessed; but the mode of treatment established here is such, that we contemplate the most formidable attack, but with little, if indeed any, apprehensions for the result. The cure of this fever is at this station almost reduced to certainty; otherwise I am satisfied, from the great intensity of very many of our admissions, and the consequent deaths likely thus to ensue, a universal panic would now be existing in the garrison, whilst encampment, fumigation, and white-washing would be the order of the day.

"I beg it may not be supposed I arrogate to myself any merit for this happy issue; I pretend to nothing but a rigid observance of the rules laid down by the admirable, and, let me say, illustrious Jackson, which, aided by the croton oil of Mr. Tegart, and which, independent of its purgative properties as a febrifuge, he justly remarks *cannot be too highly spoken of*; and seizing the first remission to administer twelve grains of quinine, for which quantum we are indebted to Mr. Corvin, hospital assistant; and, finally and lastly, to the *never-failing*, constant, zealous, and indefatigable co-operation of Mr. Greatrex, assistant-surgeon, Royal Regiment, this happy result is due."

When I reflect on the various professional conversations I have held with Mr. Greatrex on the peculiar cases that occurred of fever, and the really extraordinary powers of croton oil exhibited therein, and the confidence we therefore both felt in the combined practice set forth above—for in all and each of these were founded our strongest hopes and best expectations, founded, I say, on no fanciful hypothesis, but on the practical results daily exhibited,—I cannot, I confess, suppress a smile, though perhaps somewhat mingled with indignation and contempt, at the special pleading of Dr. Stevens, in support of his *trumpery*; and am strongly inclined, notwithstanding all he pleases to say in favour of it, to agree with Dr. Nelson of Trinidad, in reference to Dr. Stevens's hypothesis and treatment—"a practice, I am sure, he himself would not rely on in tropical fever." It is not unworthy of remark, that Dr. Stevens seems to forget the evil spirit of disguising his remedy which we are charged withal; for, in the latter part of his reply, in a whining tone he states, "they knew not the advantages they obtained from it." Now it is very possible, that the Doctor's hypothetical reasoning may have had some weight, which I am led to believe it has, with the profession at home, though rejected in the Tropics; and, alas! that such stuff should be received in London, yea, and quoted too!—though there are doubtless many there, as well as elsewhere, who are not only caught with novelty, but love to range in the wide field of medical speculation—I will not call it science—however unfounded it may prove. but for me, reflecting on the numerous theories carried into practice, founded alone on hypothetical reasoning, within the last half-century, and which have long since been abandoned and passed away even from recollection, and whose peculiar lot it has been for some years past to witness fever in all its forms, from the mildest to its most aggravated and malignant character; where promptness, activity, and decision were unceasingly demanded, and where the remedial means were not only of the *most active kind known in practice*, but employed with an energetic boldness, called for by the imminent danger of the patient, though stated by Dr. Stevens as "inert and useless," and used with a most perfect confidence, arising from their *experienced* and *well-known* efficacy in combating it; under such circumstances, can it be believed I would lend myself to an hypothesis, which, as far as I know of its principles, my reason condemned, and which, in truth, from the moment first mentioned to me by Mr. Greatrex of its humoral origin, only excited a smile, and was never after thought of by me—as I firmly believe, with all due deference to the great Dr. Stevens, that the whole series of the phenomena exhibited in fever has its *sole* and *only origin* in the influence of the nervous system generally, whilst through its ramifications particular or peculiar organs become more or less affected. It is through this system that the secreting organs cease to perform their functions, though the circulation, as has been experimentally proved, may, nay does, still continue; but thus becomes deteriorated, and travels its weary round without any replenish from those sources which, nearly or altogether ceasing to perform their functions, become, as it were, locked up. The blood thus becomes altered, and in the venous circle may become of a darker hue, though its peculiar and characteristic colour in the arterial circle continues, equally as it is found in disease in Europe, to the last moment of life. And whilst on this subject, let me say, according to my experience, the blood of all those who die of acute disease, whether in the Tropics or in Europe, or in the Canadas, or in Nova Scotia, invariably presents the same identical appearance, without the slightest discoverable difference. This recals to me the tirade Dr. Stevens indulges in on our practice, more especially the use of the warm-bath in fever, unreservedly condemning its use in *toto*, but more particularly in those stages of fever in which *he says* we employed it, though perfectly ignorant himself what these stages were; for all that he knew of its use was from Mr. Greatrex, who thus writes:—"We placed the patient in a hot-bath, at 110 degrees, and when well heated and he perspires, open a vein, and take away from 36 to 60 ounces of blood, &c." I would ask Dr. Stevens, does it necessarily follow that the access of fever in every individual is "a burning one," or marked by high excitement? Did Dr. Stevens really practically know what he presumes so didactically to write about, he would know the worst and most insidious form of fever to be met with in the Tropics, is that in which there is little perceptible excitement or little to be discovered, either from the pulse or skin, at the *onset*, but marked with a peculiar and indescribable cast of countenance, and that it is not "a burning fever," as he expresses it, yet which requires the most prompt and active treatment, to cut it short at the very commencement, far different from the kind of medicine expectante he advises; else, if lulled to security by the apparent mildness of the attack, it soon exhibits its malignant and deadly character, whilst the post-mortem examination shews to how great an extent it had carried its ravages even in the short space of perhaps 48 or 70 hours. I would not have it at all understood as being apologetic for the times or stages of disease at which we had recourse to this *invaluable remedy* in tropical fever; but to show that Dr. Stevens, notwithstanding what he asserts, could not possibly know under what particular or peculiar circumstances the hot-bath was or was not used. The objection made against this mode of practice is, it seems, according to this most didactic Doctor, that the blood was *already too red* for such treatment. How the learned gentleman discovered this I am at a loss to know; for, I nevertheless take on me to say, from experience in blood-letting, that, bleed at what stage you will in fever out of the hot-bath, the blood *never*, I may say, so very few are the exceptions, presents any difference whilst flowing in this or that stage from its natural dark venous hue, though it may present a more or less firm crassamentum, with a greater or lesser quantum of serum, when cooled, and when also occasionally is exhibited a buffy coat; but, as to its *mere colour*, I again assert, there is no deviation whatever from its dark natural venous hue, at whatever stage you may subtract blood from a vein; and, as to the colour of the arterial blood, it is always the same, be it remembered, Dr. Stevens, though you have misrepresented this fact, let us charitably suppose *ignorantly*, to the College of Physicians.

The combination of soda with nitrate of potass is doubtless, under certain circumstances, as good as *any other* saline medicine to act on the secreting organs, *when once aroused* to impression; but I fearlessly assert, of not one whit more value than any of the neutral or purging salts, either in combination or separately given; or than the abused supertart. of potass itself. Such were the views and *no other*, having no reference whatever, as far as I was concerned, to Dr. Stevens's doctrines, our ignorance of which he justly accuses us of; for, in truth, they never made the *slightest* impression on me, that led to my acceding to Mr. Greatrex's desire of prescribing this remedy at St. James's Hospital, which, as already stated by me, was frequently given; and when I admit this, having no wish or desire to disguise any part of our treatment, be it also known, in the worst and most formidable forms of fever we had to deal with, such as might be termed malignant, aye, in many hundreds of cases, the administration of this remedy was *never thought of*, much less given. Further, in my own case, in September, 1829, whilst labouring under the malignant form of fever, then epidemic, relative to which I have already given an extract of my letter to the Director General, and though in imminent danger of my life, yet not one particle of this so much vaunted remedy was administered to me. Enemata, shower-baths, croton oil, and quinine, were the routine of our practice, as already shewn, and the remedies TRUSTED to, with bleeding at the commencement, *in the hot-bath*, nearly ad deliquium. And whilst Dr. Stevens dogmatically reprobates its use, never shall I forget, though almost exanimate on coming out of it, the grateful relief I experienced from the intolerable head-ache and general pains that pervaded my whole system. In face of all this, Dr. Stevens asserts, "from my own statement, or that of the Trinidad Army Physicians, though they endeavour to disguise it, yet 'tis clearly proved, 'tis to the saline treatment they really attributed their success." Surely, if such was the case, and we had really cause to be satisfied of its potency, let me ask, can it be supposed I could be so stupid, when my life was at stake too, not to have demanded it, or Mr. Greatrex, who was my principal attendant, though visited by all my professional friends at Trinidad, not to have prescribed it for me? This one instance will speak volumes, and evince the estimation the combined soda was held in, and its classification amongst us, *merely as a very secondary remedy*.

At the very onset of what now has assumed the character of a controversy, I would desire it not to be forgotten my disclaiming entering at all into the hypothesis advanced by Dr. Stevens, considering it, as I said then, the mere ephemeral "running riot" of a vain and restless spirit for notoriety; yet a disputation on this said hypothesis is endeavoured to be fastened on me, as though I must of necessity have been connected with and participated in Mr. Greatrex's opinions, as stated by him in reference to the conversation that took place between them at St. James's Hospital, allusion to which is made in the letter he addressed to Dr. Stevens. I have now, therefore, to request it may be clearly and distinctly understood, however I may be induced, en passant, now to notice it, that in taking up my pen it was purely for the purpose of giving a plain statement of practical and anatomical facts—to inform the profession of the real state of the case—to disabuse them from the errors they might be led into by giving credence to a writer who, reckless of consequences, claimed for himself that which, under similar circumstances, I will take on me to say none of gentlemanly feeling in the profession would for a moment even have dreamt of doing;—and whilst I am accused of artfully applying, with an intention to mislead, the term "soda," in lieu of his combination of muriate of soda and potass, yet had not himself the candour, though well knowing how extensively it had been used both from our books and from the very strong testimony given of it in Mr. Greatrex's letter to Dr. Stevens, to say one single word, or make the slightest allusion to one of the most powerful, effective, and extraordinary remedies, not only in fever, but also in all those diseases in the nosology of Cullen classed under "nervous," that ever was exhibited; from which I am led to think, as well as from the splendid discoveries of late years, relative to the functions of the nervous distribution, that had that learned Professor lived in our days, he would have classed fever as an order of "neuroses," which he seems strongly inclined to have done, and not as it now appears in his works. The following is what is stated to Dr. Stevens by Mr. Greatrex, yet is withheld, *from candid motives no doubt,* from the public and the College of Physicians, when he made his memorable statements. "From the time of his waking now, or in a few hours, we administer the croton oil, agreeably to the directions published by Mr. Tegart, in the London Medical and Physical Journal, for August, 1828; and *the effects of this oil, as they have appeared in some hundreds of cases* which have come under my observation in Trinidad, *fully support the high merits which Inspector Tegart has assigned to it.*" Let us go no further even than this one passage, and let me ask if it is not sufficient in itself to stamp Dr. Stevens's want of candour and fairness? at all events it cannot fail to establish his insatiable vanity and egotism;—and notwithstanding the clearness and evidence of the above passage, the gentleman still supports his unqualified assumption in the commentary he makes, in reply to my statement to you, on Mr. Greatrex's letter, though his pretensions are not in one single instance supported by the letter itself, notwithstanding all he had himself said at Trinidad in favour of his mode to the latter gentleman. He, it would appear, was inclined to favour these doctrines yet, with the fairness and candour of an honest man, after telling him he had administered the remedies as advised, continues—"but with what *particular effect,* I regret to say, *I cannot determine* for you, in consequence of its being *mixed up with the effects* of *several* other measures which we are in the *habit* of *adopting.*" Not one word of marked beneficial effect (yet the reverse is published,) have we here stated on Dr. Stevens's behalf; whilst, on the contrary, let it be observed, a marked and decided preference is clearly given in the foregoing eulogy relative to croton oil—yea, "having been tried in some hundreds of cases, and fully supports the high merits which has been assigned to it, &c." and let it be noted, for I know the fact, that these very "hundreds" above quoted are those which Dr. Stevens has asserted were cured by his nostrum. Again, as regards quinine in large doses, "we were led to this practice by a medical officer telling us it was given in that quantity on the Coast of Africa with *the best effects,* and *such have been the effects here.*" Mr. Greatrex, after adverting to Dr. Jackson's modus medendi, which it will be observed we had adopted, and which he thus introduces—"The way *we combat* fever *is agreeably* to Dr. Jackson's *system,* so admirably set forth in his invaluable work,"—and thus concludes, "under Divine permission, the *above system* (the principal and leading features of which he had detailed with a favourable commentary) has been applied to 340 cases or thereabouts, &c. and with such success, that, during the last seven months, not a case died." Let us here pause, and ask, would any man, with any sense of propriety or of feeling, or common decency, apply, much less publish, from the foregoing quotation, the "system," the integral parts of which are respectively so highly spoken of, exclusively to himself? when the best, after all, it will be seen, that is said of Dr. Stevens's recommendation is, *most dubious;* for "he regrets, with what particular effect he cannot determine, because it is mixed up with various other remedies, &c."—to which, let it be observed, are given the most marked expressions for the beneficial effects, *according to recommendation,* THEY *are found to produce.* Surely with some propriety, and I do think most deservedly, might Mr. Tegart lay public claim to the merit of successful treatment, whilst even with him it might in some measure, and with some little shew of justice, be disputed by the gentleman who recommended to us the powerful auxiliary of *large* doses of quinine in lieu of the more minute and frequent ones we had been in the habit of giving, were they of the same temperament and monopolizing spirit that so eminently characterises the author of "Observations on the Blood;" and whilst I admit a full measure of credit to both these gentlemen for the benefits we derived from their recommendation, yet I much doubt if any plan of treatment in tropical fever can ever be remarkably successful, unless aided by the exertions of a *most watchful* and intelligent medical officer.

I have stated two instances, in which the garrison was unhappily deprived of this assistance, and the consequence that appeared to me to arise therefrom; but the reverse of these I ever found Mr. Greatrex, and to his exertions, aided by croton oil and quinine, the subsequent success must be attributable. And here I would ask the profession to contemplate the well-known powerful effects on the constitution of either of these remedies, whether given separately or in conjunction, as we often administered them; —when this is considered, let them say, can we be blamed for calling Dr. Stevens's pretensions *presumption*, or class *his* muriate of soda and nitrate of potass but as the *veriest trumpery*, since the days of Perkins's metallic tractors, that ever has been attempted to be pawned on the medical public as a wonder-working remedy in fever? Under "Divine Providence," the recorded system however, of which both the foregoing stated remedies formed with us the *most marked* and *prominent features*, and which, let it be always remembered, *were never omitted to be administered to a single case of the* 340 *alluded to*, was, notwithstanding Doctor Stevens's assertions to the contrary, pre-eminently successful.

Doctor Stevens asserts, that my dissection reports entirely agree with the appearance of the blood he has stated. This is indeed assertion with a vengeance. The fact is, seeing the absurd and unsupportable ground he had taken, he now comes round to my statement, gilded over with that special pleading, apparently so congenial to him, and, as it were, forgetting (and, I dare say, for his own credit, sincerely wishing it to be forgot) "the dark watery black ink blood" so piteously and poetically previously expressed as occupying *alike* both circles of the vascular system, states now, in allusion to one of them, that 'tis dark, grumous, nay, he would say, viscid—but is perfectly silent as to the assertion he was pleased to indulge in about the contents to be found in the arterial circle.

As to the graphic portrait of the fever he has favoured us with, it seems now it is but a solitary exception. Exceptions, we are taught to believe, only strengthen a general rule or description, such as I have given; yet, from this solitary exception alone, if ever such existed but in his *imposing* imagination, Dr. Stevens comes forward with an hypothesis, kindly to enlighten the profession! "ready cut and dry," to meet all exigencies, and all classes of tropical fever. He disdains misrepresentation or want of candour. Here again we are at issue, and here again I arraign him of both. I have already pointed out his want of candour relative to Mr. Greatrex's letter, and now accuse him of knowingly, wilfully, or albeit ignorantly, misrepresenting tropical fever generally, and doubly of misrepresentation, contained in the following quotation:—"In both cavities of the heart the blood is equally black, and in the whole vascular system all distinction between arterial and venous blood is entirely lost:" and again, "the colour of the whole mass of blood, both in the arteries and veins, was changed from its natural scarlet or modena red to a dark black." This is not a fact; I am bound to say, it is *absolutely* and *utterly without foundation*. There is no blood in the arteries after death in tropical fever cases; consequently it is a mere flourish of the learned gentleman's to introduce his hypothesis, which I should be inclined to liken to the flourish or clash of a high sounding orchestra to introduce a new pantaloon in a new pantomime—to say that all distinction between the arterial and venous circle is lost, and that the blood is changed from its natural colour to a dark black. In the dead subject, in this disease, I repeat that the arteries are invariably empty. I repeat this not only on my own authority or observation, but on that of all the tropical practitioners I have conversed with on the subject. I also deny *clearly*, *distinctly*, and *positively*, that any change from its natural scarlet characteristic takes place in the living artery *in the progress* of fever. Conversing with Dr. Musgrave, a most respectable practitioner of Antigua, and not unknown to the profession, on this most strange assertion, sent forth to the public, he reminded me of the case of an officer of rank belonging to that garrison, to whom he was called in, and who died of malignant fever a few days previous to my arrival there, and whose temporal artery was divided an hour or two before death, yet the arterial blood was in no way different in hue from its natural colour in health.

To advert to Dr. Stevens's boasted hypothesis: and again, I say, alas! that such sorry stuff should occupy the attention or be food for debate in the profession—whilst he lays it down that the blood owes its red colour to the salt it contains, as well as its preservation, "in common with the waters of the deep, owing the power of self preservation to their saline impregnation;" and states that death takes place in tropical fever from the blood being deprived of its saline constituents, nay, adduces as positive proof of this, "the dark black ink-like appearance of both the arterial and venous blood." Here again we are at issue; for we have shewn the *absolute reverse* of this assertion is the *real fact*—that the colour of the arterial blood, from the commencement to the finalé of fever, whilst blood is contained in that circle, preserves its own peculiar scarlet hue, fully establishing, in my mind, the commonly received opinion of the blood owing its florid colour to its transmission through the lungs;—and as to the blood generally being in a state of apparent dissolution, I would ask how it can be otherwise? When secretion mostly, if not altogether ceases, it is then deprived of the pabulum vitæ, and must eventually become deteriorated and unfit for the purposes of supporting life. I shall now add that, until the learned gentleman proves, by actual *bona fide demonstration*, the truth of his assertion, in contradiction to what I have, in common with all other tropical practitioners, noted and seen—"that all difference between arterial and venal blood ceases, and that both alike assumes the watery dark black *ink* colour in the progress of fever," which, be it remembered, is the "head and front" of his hypothesis—the whole baseless fabric must fall to the ground, even in the estimation of the most credulous; and, till he demonstrates—for, let it be remembered that, if true, it is to be demonstrated—mark, I say, *demonstrates*, before credible witnesses too, on the living subject, the unqualified and most extraordinary assertions made to the College of Physicians and the public generally, the profession can be at no loss to appreciate either the correctness of his statement or the immediate views that dictated them.

To revert again to the "art" Dr. Stevens discovers in my using the term "soda," &c. I utterly disclaim the accusation. I made use of the term of the *base* for his compound without really any precise or defined intention, more than to mark, perhaps, a certain degree of contempt for the boasted hypothesis, and the remedy founded on it, and so vaunted as a specific in fever, its author giving in support of it *imaginary*, at least, let us say, dissection reports, assuming therefrom false premises, and arguing from them as *established* facts. Let it also be remembered, however designated occasionally by me, that the actual composition of his "sodaic mixture" was fully stated: and, as "a rose will smell as sweet by any other name," I fancy the virtue of his nostrum was not rendered the less potent by the term I happened to give it! I have, therefore, no right to be taxed with unfairness or art on that account. In the "display of my deep research," I recently availed myself of the published opinion of Dr. Paris, on the *principle* of curing disease through the medium of chemical action, or, now let me add, mechanical action on the blood; for I consider the *principle* of both exactly alike—a *principle*, be it remembered, as old as the hills; and a principle, be it also borne in mind, abandoned, because of the mortality produced by acting on such a truly fanciful hypothesis; yet, I might almost say, this very identical *principle*, (for the one is to destroy by chemical agency the presumed excess of *a something* the blood might have become injured by; the other mechanically to supply the deficiency of a constituent, equally presumed, it has become deficient of) in fact, the humoral pathology is at this time of day, after the quietus of a century, brought forward to give eclât and celebrity to its author!

Having, I trust, fully replied to all Dr. Stevens has advanced in support of his pretensions, it now remains for me to remark on the "God bless him for it" evidence of the letter brought forward to substantiate the learned gentleman's claim, and, without meaning to advert to the folly of calling in vulgar opinion as corroborative of medical success in the treatment of disease, using that term to designate those who give opinions on professional points with which they are totally, and, I might say, necessarily unacquainted, but knowing the person alluded to, to be "a plain blunt man," whose habits, since I have known him, lay diametrically opposed to professional rules or pursuits, and who had nothing whatever to do with the hospital, or the treatment of its inmates, or, in truth, interested himself *in any way* practically on the subject, I am at a loss to know how such a person's testimony could be supposed corroborative proof, "strong as holy writ," to substantiate the pretensions of Dr. Stevens, adduced, too, as an upsetting proof to what we have advanced as our chief stay and principal prop in tropical fever. To point out the great inaccuracy of this kind of evidence on a mere numerical fact, in which one would suppose there could be no mistake, the writer says, "tell Dr. Stevens since he was here (now four months yesterday) the Royal Regiment have not lost a man;" whilst it will be seen, from the returns I have already stated, we had actually lost five within the same period! So much for the accuracy of this description of evidence. I however made it my business, in consequence of the above, to wait on the officer alluded to at Morne Fortune, when lately at St. Lucia, and spoke to him of the quotation Dr. Stevens gave of his letter, wherein was given unqualified praise to that gentleman for the successful treatment of fever, &c. he smiled at the learned gentleman's credulity, and said, "why I took Dr. Stevens to be a man of sense, but what a bl——d he must be to quote me, or introduce me at all in his publication, for any thing I may have said or written on a subject I really knew nothing about. I knew nothing, he continued, of what was going on in the hospital, or the treatment of the sick there." But it seems, said I, notwithstanding, you wrote to some person, giving to Dr. Stevens all the credit for the cure of fever at Trinidad. "Why," he replied, "you may remember, on or about the time of Dr. Stevens's visit, we became healthy; and hearing something of a new plan of his for treating fever, I took it into my head, that he might have given some hint to Greatrex, but I really knew nothing whatever of the hospital practice, and had no particular meaning in what I wrote." It is a pity, in despite of what he had himself from the best source, Mr. Greatrex, that Dr. Steven's temperament disposes him so easily to construe, in his own favour, whatever may be written, or so greedily to swallow up, no matter from what source, whatever is flattering to *self*. It appears the Doctor was predisposed to this "sweet unction," and therefore willingly lent himself to the delusion, giving a broad interpretation to what, at best, was merely meant by its author as a French compliment. I imagine it will however serve as a lesson to the officer alluded to, to be more particular in future, and not pass hasty opinions on subjects, under his hand too, of the merits of which he must needs be totally unacquainted, and of which advantage may be taken by those whose delicacy may perhaps be rather obtuse when self is concerned.

There seems to me a *tinsel* display of scientific research strikingly observable in the history of the Doctor's hypothesis, which I am induced here to take notice of, however foreign it may have been to my previous intention to do so. Even in the prefatory page of his "Observations on the Blood," we find it in the dissection report he has indulged us with, the most minute examination, it seems, of the *ablest* anatomist of those who died of *his fever*, could not detect any lesion of structure in any part sufficient to account for death, till, says he, "we examined the state of the once vital fluid, which, in both cavities of the heart, is equally black, and in the *whole vascular system* all distinction between arterial (ay, in the dead subject too!!! credat Judeus) and venous blood is lost." Here, then, by this scientific author's account, the enigma is at once solved, and thus these fluid appearances in death are made to explain the cause of death itself. The secret is now out—from the acute discernment of the learned gentleman's discovery, with perceptions peculiar entirely to himself, and not within the pale of vision of the whole of the tropical practitioners, that not only the veins, but even the arteries! are full of watery, thin, black, ink blood. From such premises as these, let it be recollected, he states he arrived at his conclusions, and henceforward proceeded to the application of his means, to renovate the dark, black, watery, ink blood in the arteries;—for I may be allowed, as the gentleman "so clearly" and so unequivocally states all distinction ceases, to give a part for the whole of the circle—to give it consistency, and furnish it with the *presumed* saline properties he conceives or asserts it had lost, and thus, in fine, to cure fever. This, I believe, is the "cream and marrow" of what the learned gentleman advances, which being, he states, done at proper time and season, the blood is regenerated, the fever routed, the cure made perfect, yea, proved, he says, in some hundreds of cases in the West Indies, equally, I imagine, *as well authenticated* as those I know myself of, and quoted to the public from Mr. Greatrex's letter. As to the resemblance "of the waters of the deep owing their self-preservation to saline impregnation" in common with the blood, we must say it is a gratuitous assumption at best and unworthy of notice; for we have no means, chemical or other, to detect any thing saline in the waters of the deep lakes of fresh water common both in Europe and America; therefore this assumption, in comparing an animal fluid, like the blood, to sea-water, must be taken for as much as it is worth—nothing. By the bye, this reminds me of the "ingratitude" we are accused of in not acknowledging the debt we owe to Dr. Stevens, for giving us this *bright* idea, as a beacon to guide us in practice, and also for "so clearly seeing and pointing out to us the cause of our mortality in fever." The want forsooth, or diminished quantum of sea-salt in the blood, which he says, being replenished by being taken at proper times and seasons, by its colouring powers and renovating properties, banishes disease and saves life. This he elucidates by the so often repeated black watery ink blood, found, he asserts, in the whole vascular system, but which, alas! for the stability of his doctrines, is but a mere assertion, unfounded alike in truth and in fact; and I must say, whilst his hypothesis rests on this wretched baseless foundation, that no credence whatever can or ought to be given to any treatment of fever founded on it. I am inclined to think, with better pretension, I might retort the charge of ingratitude on the learned gentleman himself, for his perfect silence, and noticing in no way whatever, my setting him right in his report of the state of the fluids, of what is *actually to be seen* in the progress of fever, and what *is not to be seen* in death, in shewing the empty state of the arterial circle on the one hand, and the blood in the venous *diametrically the reverse* of *what he states* on the other.

There is a strange inconsistency, too, relative to fever, in the work this gentleman has indulged the public with, observable from first to last of the publication. In the beginning will be found—"there is no excitement sufficient to injure the solids." As we proceed, we find—"when proper means are used to protect the organs from the *increased excitement* during the early stage of disease, and after excitement is sufficiently reduced, when proper nourishment is given, and *certain saline* medicines are *timely* and *judiciously* used, the bad symptoms are prevented;" and consequently no man can be so qualified—happy news for the good folks of England, who actually, at this present moment, possess *such a treasure* amongst them, to give them certain salines, *timely* and *judiciously*, as the erudite writer himself! Now, I would ask any tyro in medicine, what is really all this we read, particularly the context of one part of this work with the other but *verbiage*, yea, *downright verbiage*. The writer starts, let it be remembered, as a disciple of the humoral pathology, and introduces a *fancied* case of tropical fever to support it; and terminates apparently by being a solidist, talking of "protecting the organs from the increased excitement:" and, again, "after the excitement is sufficiently reduced, and proper nourishment given." Without further noticing the discrepancy so apparent, I would ask, who that knows any thing of fever, or its treatment, would prescribe any nourishment, properly so called, until the excitement had nearly or *altogether* abated? then, and not till then, the secreting organs have commenced their respective offices—then, and then only can the stomach receive, with impunity or relish, food—ere this takes place, food or nourishment, beyond the merest slops, in small and very minute quantities, should not be given; otherwise it oppresses the stomach, acts on it as a foreign body, and consequently tends to increase excitement, and to add to the already existing evils: and again, for the sake of common sense, divested of all the affectation of professional jargon, what, I would ask, is the reduction of excitement but the abatement of fever? The morbid chain of disease is now broken, the cure "progressing," without too "the timely application of certain saline medicines;" for it is evident the nervous energy heretofore impeded has become renewed, the glandular system has commenced its natural functions, the pabulum vitæ is thus furnished to the nearly exhausted circulation, and let it be ever borne in mind, the stoppage of healthful secretion is the leading feature of *all febrile* affections, whether tropical or otherwise, and therefore demands our most particular attention; for herein, I fearlessly contend, is "the head and front of offence" immediately indured by the frame *generally*. Is it then to be wondered at, that the blood should participate in the contamination engendered all over the "mortal evil," and suffer serious derangement? I repeat, in all its constituent parts, when without any perhaps, or at most a very scanty replenish from the secreting organs it went its "weary round" of the body.

A service of seven years in the West Indies (a regular transportation) has taught me that whatever hypothesis or treatment of fever, it pleases any man to indulge in, his success in its treatment will always depend on the importance he attaches, and the ratio of attention he pays to the secretions, as a primary object. When the practitioner rigidly attends to these, I do not mean by constantly prying into night-chairs or chamber-pots, with a *seeming* learnedly scrutinizing examination at each returning visit,

(indeed I have often, I confess, been struck with the ridiculous resemblance of the sapient curious look exhibited by some professional men I have met with in life, examining "the loaded vase," to that of a magpie peeping into a marrowbone; for both seem, with the same meaning, to look, and chatter, and pry again) for I contend, that the reports of an intelligent attendant on these degrading and disgusting examinations, are, in ninety-nine cases out of a hundred, quite sufficient, together with the tact common to most practitioners accustomed to treat disease, to inform him, in conjunction with other striking appearances, such as, the countenance of the patient, a grand desideratum in practice, aided by what the tongue presents, and feel of the skin, and actual posture of the body, that he has or has not yet attained his object; and therefore, I say, *faithfully* indicate his future proceedings. For I must say, I do think there is often as much quackery, and as much attempt, by many, at scientific imposture in the examination of a filthy night-chair, as in any other legerdemain, let me term it, of the profession; and I maintain, that it may well, *generally*, and without injury to the patient, be discarded from practice. But to return from this digression, when the practitioner directs his whole attention to arouse the secretions, I care little where or in what tissue or fluid of the human frame it pleases him to give the disease "a habitation or a name." His hypothesis, or theory, or learned fancy, or whatever else it might be called by him, thus becomes harmless; let him therefore indulge his folly, and lodge fever wheresoever it pleases him, and most learnedly descant on *the why* it should be there, whilst all his practice tends, and is entirely directed to arouse healthy secretions, by the administration of such remedies known by experience to act powerfully on these organs, though given professedly to neutralize this in the fluids or, colour that, either renovating the quality or reducing the quantity of some noxious *something* the blood is presumed to contain. With views entirely directed to the secretions, we acted as being solely affected by the nervous influence, and the well known powerful effects of the remedies we employed to these ends, will amply testify the fact; yet in defiance of all this it is asserted to the world, that a two-penny halfpenny remedy, and the appearance of the blood, pointed out by Dr. Stevens, which it will be seen exists too but in his imagination, was the cause and origin of our practice and success!

To the vulgar taunts throughout Dr. Stevens' reply, and no less vulgar threats contained therein, asserting that "it *could* be proved that Dr. Hacket admitted every syllable to be true which I have stated in my paper;"—I am at a loss in what exact language to reply; I shall, however, but merely say, I repudiate the assertion with the scorn and contempt it merits, and *defy* Dr. Stevens to the proof. I never classed, or spoke, or thought of his remedy as possessing higher powers than any other of the saline substances I have already stated, and never considered it more than the merest auxiliary, seemingly not ill adapted to keep up secretions *when once aroused*—even if I ever did say so much; and I call on Dr. Stevens now to give his threatened "proof." The fact is, that though the learned gentleman calls his remedy a non-purgative salt, and thus gives it, as he states, to act mechanically on the blood, we estimated its value on the other hand, only by its action on the bowels and secretions generally. With this view, in my first exposé it is stated, that a combination of the muriate and *carbonate of soda* with the nitrate of potass was administered to the sick, which was found, under the circumstances I have stated, to keep up the action, generally, of the viscera; from this it will be seen, we dared to act on a sound theory, and that neither the hypothesis or remedy of Dr. Stevens was, *after all*, attended to: yet this, it seems, is the reason my paper should not have been published! that Dr. Stevens might be allowed to continue to hoodwink the profession by statements founded neither on anatomical or practical facts. Soaring in the region of fancy, he mixes salt and blood together in a porringer, and from the hue thus produced, argues from analogy that the same takes place in the living system! Can there be any thing more fallacious than likening appearances drawn from such premises to the living fluid *within* the miraculous and really wonderful laboratory of the human frame? yet such, at least, so he states, is pronounced the greatest discovery ever made in medicine. Now, if rendering the peculiar deep hue of the venous blood of a scarlet colour, be proof of saline existence in it, and essential to the cure of fever, I well remember, when a student in a syphilitic hospital, how frequently this appearance presented itself when patients under the influence of mercury were blooded: indeed, I have not only started myself at the first gush following the lancet, of the seemingly arterial blood that flowed, but witnessed the same surprise in my fellow students, till the frequency of the occurrence reconciled us to the fact. I have also seen since, though not in the tropics, this self same effect exhibit itself in fever cases, treated by mercury, when the individual appeared evidently under the influence; from this I am led to imagine, if Dr. Stevens's hypothesis has any foundation in truth, its proper proof rests on the *perceptible* effects of his saline remedy on the living fluid, contained within its own proper vessels, and not by an admixture of salt and blood in a porringer. There can be no difficulty, I imagine, in testing, at least its colouring powers in disease, by adopting this saline treatment; and when the cases are presumed to be sufficiently saturated with this all-renovating and all-colouring remedy, the lancet will then *demonstrate* the truth or error of the statement, with the concomitant of bona fide convalescence. To me, something of this kind seems imperative to be done, if *the system* has really a foundation to rest on; for the ablest chemists heretofore could throw no light, by experiments on the blood, on the nature of different diseases; and although some of our first and best chemists contend that it is on a peculiar animal principle the colour of the blood depends, many, however, equally eminent, assign it to another cause: but the Stevens Sidus has "clearly elucidated the truth by the greatest discovery ever made," and fixed it on the immutable principle of sea-salt!! which we call on him now to substantiate equally by experiments on the living, as he has attempted to do on the dead fluid.

I shall now finally state, in answer to all Dr. Stevens urges in his communication, and to shew the profession generally of how much value in practice his doctrines were and are yet considered in the treatment of tropical fever, that, as far as I could learn, throughout all the following islands, which I lately visited on duty, there is not one convert to his opinions, and that the practitioners generally throughout them, respectively, cry "pshaw!" both to his writings and pretensions at Antigua, Dominica, St. Lucia, Barbados, Tobago, Trinidad, Grenada, St. Vincent; in the next place, for the last fifteen months that I had been stationed at Antigua, *not one particle* of his "sodaic mixture" was administered to a *single fever case,* and yet our practice had been equally as successful there as at Trinidad; and, although it must not be disguised but that there is a very material difference in the two stations, as regards either the frequency or intensity of fever, yet I believe "English Harbour," which was my immediate residence, is as well known amongst the medical profession, particularly the navy, for furnishing as heavy a mortality, both amongst strangers and natives, as any other part, be it where it may, amongst the Tropics. "The Ridge" is considered the most healthful situation; this part of the garrison is occupied by the 86th Regiment, nevertheless, from amongst these we have occasionally as heavy and severe cases of fever as I have ever witnessed at Trinidad; having premised thus much, I shall now quote from surgeon Cunningham's (of that corps) last report, as the means of disabusing the community of the twaddle, wilfully or ignorantly attempted to be imposed on them. Let me remark, Mr. Cunningham is an old and most respectable medical officer, and not likely to be swayed, as hinted at by the learned gentleman in respect of Mr. Greatrex, contrary to his judgment by any man; he has now been upwards of five years in the command, a portion of which he served in Trinidad, therefore may be considered no despicable authority in the present affair.

"Five cases of remittent fever were treated in the Regimental Hospital during the quarter. Two cases were also treated in quarters, of peculiar severity, viz. one officer, Major Gibson, of the 86th—the other the wife of the Serjeant-Major. These were, in particular, aggravated cases, attended with intense degree of gastric derangement at the commencement of the attack. In the whole of these cases, the beneficial effects of the croton oil, in suddenly and effectually arresting the violent irritability of stomach, and in alleviating the urgent febrile symptoms, were particularly apparent; the quantity administered was four drops for the first dose, assisted by active purgative enemata and warm bathing, or the cold shower-bath—the latter was found the most beneficial; a second dose of the oil, to the extent of one-third to two drops, was in some of the cases found necessary. The sulphate of quinine, in doses of from 10 to 12 grains daily, was had recourse to at as early a period as circumstances would permit. Venesection was in none of the cases had recourse to; no instance of protracted convalescence occurred. There is every reason to conclude that, had the excessive irritability of stomach not been arrested by the use of the croton oil, and had the symptoms been combated, as formerly, by the use of other remedies, &c. black vomit, with exhaustion, must have soon supervened, with a fatal termination."

I shall here detail a case, given also in my Quarterly Report, not only corroborative of Mr. Cunningham's opinion, but strikingly illustrative of the almost, let me say, miraculous powers of croton oil in the worst forms of tropical fever.

"I was solicited, by message from his medical attendant, to visit the parochial schoolmaster of English Harbor; being occupied, I could not attend. In about an hour, I received a second pressing message from the man's wife, to entreat I would see her husband, as the doctor declared, on departing, that he could do no more than what he had already done for him; she herself adding, 'if he did not get speedy relief he must die.' On going, I found the man had been ill of fever for three days, and, for the last fourteen hours, had laboured under irritability of stomach and almost incessant vomiting; the bowels were torpid, though purgatives and lavements had been given, and, indeed, in justice to the Doctor, I must say, the usual routine in tropical fever had been in no way neglected; there was great exhaustion—no pain—no complaint, but of constant vomiting—the skin was moist and clammy, from the violent exertions the patient was obliged to make. Here was the exact case I ever found croton oil pre-eminently useful in—I gave four drops immediately in a tea-spoonful of syrup: in a little time this seemed to increase the irritability, which is ever the case; but this soon subsided. A large bucketful of lavement was made, of salts and ol. ricini, of which four large syringes full were given promptly, and as quickly in succession as they could be administered. No operation took place, although a respite was now given the patient for about a quarter of an hour. Four more syringes full, similar to the former, were now given, when the bowels began to act; the effect produced astonished all around, both as to the excretion and quick relief obtained; suffice it to say, in about two hours the severity of its effects began to subside, the irritability of the stomach was nearly gone; the patient became tranquil, complaining only of great weakness. As we advanced (the irritability of the stomach having for some hours subsided), a ten-grain dose of quinine was given, and repeated the following day. Convalescence was now established; in a few days, health was restored." I recapitulate this merely as Trinidad practice:—Away, then, with the most groundless and unfounded assumption of Dr. Stevens, in his "Observations on the Blood," that we cured fever in that garrison by the soda combination, forsooth, of his recommendation.

Still further corroborative of what I have advanced, as regards the confidence reposed in croton oil and quinine, I herewith send you a few letters received from medical friends at Trinidad, to whom I addressed myself on the subject. You will be pleased to give to these such publicity as you may deem necessary.

I shall now take leave of Dr. Stevens and this controversy altogether, declaring—

"I'd rather be a dog, and bay the moon,
Than such a Roman,"

and shall take no further notice (except it comes in a more tangible shape than assertion, for assertion and proof seem synonymous in the Doctor's vocabulary), of whatever his singular self-sufficiency may again prompt him to state to the public. In taking leave, however, of this gentleman, I cannot, I conceive, do better than give him the following theme, taken from a modern writer, for his cogitation and application. "There is nothing better known to those who are conversant in medical practice, than that the most ignorant and the most shallow, those of the least learning, nay, those of no learning at all, are the most addicted to hypothetical reasoning—the most infested with presumption and conceit."

Should Dr. Johnson do me the favour to publish this communication, the whole statement will then be before the profession generally throughout Europe and America; to the candid and liberal of whom, in both countries, most willingly do I submit the cause of *truth*, which, and a sense of what is due to them and the public, could alone, I assure them, have induced me thus to come forward (however feebly, perhaps, I may have executed my task) in its advocacy; and should I in any manner seem to have exceeded the bounds of courtesy, beyond what the novel peculiarity of the subject demanded, I confidently claim their indulgence, and would intreat them to consider it in its true light —the *natural consequence* arising from an *highly-exciting cause*.

I have the honor to be,
Sir,
Your most obedient Servant,
W. HACKET, M.D.
*Surgeon to the Forces.*

*To Dr. James Johnson, &c.*

Printed by G. HAYDEN, Little College Street, Westminster.

